# Medical, Technical and Audiological Outcomes of Hearing Rehabilitation with the Bonebridge Transcutaneous Bone-Conduction Implant: A Single-Center Experience

**DOI:** 10.3390/jcm8101614

**Published:** 2019-10-03

**Authors:** Faris F. Brkic, Dominik Riss, Katharina Scheuba, Christoph Arnoldner, Wolfgang Gstöttner, Wolf-Dieter Baumgartner, Erich Vyskocil

**Affiliations:** Department of Otorhinolaryngology, Head and Neck Surgery, Medical University of Vienna, Währinger Gürtel 18-20, 1090 Vienna, Austria; faris.brkic@meduniwien.ac.at (F.F.B.); dominik.riss@meduniwien.ac.at (D.R.); katharina.scheuba@meduniwien.ac.at (K.S.); wolfgang.gstoettner@meduniwien.ac.at (W.G.);

**Keywords:** Bonebridge, transcutaneous hearing implant, bone conduction, complications, audiological outcomes

## Abstract

Bone-conduction implants are a standard therapeutic option for patients with conductive, unilateral, or mixed hearing loss who either do not tolerate conventional hearing aids or can benefit from surgery. The aim of this study was to evaluate long-term medical and technical outcomes, and audiological results with the Bonebridge transcutaneous bone-conduction implant. This retrospective study included all patients implanted with a bone-conduction hearing implant at a tertiary medical referral center between March 2012 and October 2018. Medical and technical outcomes included the mean length of implant usage, medical and technical complications (skin and wound infection, lack of benefit, technical failure), explantations and revisions, coupling approaches, implant failure rate, implant survival and the implant loss for added follow-up years. Auditory results were measured by functional hearing gain and the Freiburger monosyllabic test at 65 dB sound pressure level. Sixty-four patients were included in the study; five of these were implanted bilaterally (69 devices). Five unilaterally implanted patients were lost to follow-up. The mean follow-up was 27.1 months (range: 0.2 months–6.3 years). The mean implant usage was 25.9 months (range: 0.2 months–6.3 years). Fifty-seven implants (89.1%) were in use at the end of the follow-up period. Complications occurred in six ears (9.4%). Five implants (7.8%) were explanted without reimplantation. Device failure occurred in one implant (1.6%), which was possibly caused by recurrent head trauma. The rate of implant loss due to technical device failure (damage to device) was 1 per 72 follow-up years. The mean improvement on the Freiburger monosyllabic test (52.1%, *p* = 0.0001), and in functional hearing gain across frequencies (26.5 dB, *p* = 0.0001) was significant. This single-center follow-up reveals the medical and technical reliability of a transcutaneous bone-conduction implant for hearing rehabilitation because complication and revision rates were low. The majority of patients still used the device at the end of the observation period. Implantation resulted in favorable hearing outcomes in comparison to that of unaided conditions. Cautious patient selection mainly regarding co-morbidities, the history of chronic otologic diseases and proper surgical technique seems to be crucial in reducing complications.

## 1. Introduction

Bone-conduction implants (BCI) are a standard therapeutic option for patients with conductive, unilateral, or mixed hearing loss who either do not tolerate conventional hearing aids or can benefit from surgery. Over the last few years, implants have also been extended to single-sided deafness (SSD), by routing the signal through the skull from the contralateral deaf to the normal-hearing ear [1].

The Bonebridge (Bb) (MED-EL, Innsbruck, Austria) active transcutaneous BCI was developed to circumvent typical postoperative complications found with percutaneous systems, such as high infection rates, fixture losses, the need for revision surgery and skin dampening effects [2,3,4].

As noted by Sprinzl et al. [5], the Bb incorporates a sound processor that delivers the sound energy through the skin over an inductive link to an internal coil. The signal is then conducted to the demodulator. After processing, the signal is transferred to the implantable portion–the bone conduction floating mass transducer (BC-FMT). The BC-FMT converts the signal into mechanical vibrations, which are transmitted to the mastoid bone through the cortical fixation screws. The surgical procedure of the Bb implantation was explained thoroughly by the same author [5]. To summarize, a 4 cm long incision is performed 1 cm behind the external auditory canal. A raspatory is used to expose the cortical bone of the mastoid in order to prepare the periosteal pocket. A bony well for the BC-FMT is then carefully drilled. The coil part of the implant is placed into the periosteal pocket, while the BC-FMT is planted into the bony well. Two screws are then used to secure the implant.

Various authors have assessed hearing rehabilitation outcomes with the Bb. However, these studies mostly reported on audiological outcomes in a small number of patients. The largest cohorts included 38 [6], 28 [7] and 26 [8] patients. Riss et al. analyzed indication criteria and audiological outcomes in 24 Bb implantations [9]. To date, there have been no studies reporting on both the medical and technical long-term performance, as well as the audiological results of the transcutaneous BC implant in a large cohort of patients in a single center. As the technology is relatively new, long-term data on implant reliability is scarce. Only a few long-term studies containing revision and explantation rates due to medical or technical reasons have been published. Only one report has assessed the surgical and audiological outcomes of 20 patients implanted with the Bb device [10]. Therefore, it is still unclear if the implant provides stable long-term outcomes.

The aim of the current study was to analyze all consecutive patients implanted with the Bb device in a tertiary referral medical center over a 6-year follow-up period. Long-term medical and technical outcomes including the mean length of implant usage (in months), medical and technical complications (including skin and wound infections), lack of benefit and technical failure were analyzed. Furthermore, revisions, reimplantations, explantations, implant survival and implant loss for added up follow-up years were assessed. Postoperative audiological benefit was also evaluated.

## 2. Experimental Section

### 2.1. Patient Characteristics

This was a retrospective chart analysis of all patients implanted with the Bb at the Department of Otorhinolaryngology, Head and Neck Surgery at the Medical University of Vienna, between March 2012 and October 2018. Some patients that had been implanted with the Bb in our department and initially reported on in previous studies (Riss et al. [9], Vyskocil et al. [6]) were also included in the current study. Pediatric patients implanted for atresia and SSD were analyzed as well.

### 2.2. Surgical Procedure

Indications for Bonebridge implantation included conductive or mixed hearing loss with bone conduction thresholds lower or equal to 45 dB HL. All surgeries were performed by the senior co-authors (W.G. and W.-D.B.). Data on long-term medical and technical outcomes, including the mean length of implant usage (in months), medical and technical complications (skin and wound infections, lack of benefit, technical failure), revisions, reimplantations, explantations, implant survival and implant loss for added up follow-up years were retrieved using patient medical charts.

### 2.3. Audiological Assessment

Audiometric tests were performed in a sound-proofed room with routinely calibrated audiometers used in the clinical routine. Pure-tone audiograms and speech tests were performed prior to surgery and with the same settings postoperatively. The functional hearing gain (FHG) was calculated by comparing unaided free-field thresholds to thresholds with the Bonebridge activated. The contralateral ear was covered in all cases using earmuffs (Peltor Optime III: 3M, St. Paul, MN, USA). Audiological testing was performed preoperatively, followed by postoperative measurements taken on a monthly basis for the first 6 months. For our analysis, the last available measurement was used. Testing included the sound-field (SF) pure-tone PTA4 thresholds (0.5, 1, 2, 3 and 4 kHz). Functional gain (FG) was calculated as a difference between unaided postoperative and Bb-aided PTA4 thresholds for patients with atresia and combined hearing loss. Due to inadequacy, FG was not calculated for patients with SSD. One expert noted that traditional FG measurement was not applicable in patients with combined hearing loss implanted with bone-conduction implants and recommended calculating the effective gain in these cases [11]. By definition, the effective gain is the difference between bone-conduction thresholds and aided thresholds and therefore may be negative. Speech intelligibility in quiet backgrounds was assessed using the word recognition score (WRS) of the Freiburger monosyllabic word test, which is a test for adult German-speaking patients. Unaided and thresholds with the Bb at 65 dB sound pressure level were compared.

### 2.4. Statistical Analysis

The statistical analysis was performed with the Statistical Program of Social Sciences (SPSS: version 23.0, SPSS Inc., Chicago, IL, USA). The statistical significance (Alpha) was set at 0.05, two-tailed. Comparison of complication rates between the two FMT coupling groups was performed using the Chi-Square test. In order to compare unaided and b-aided SF PTA4 thresholds and word recognition scores, a paired t-test was utilized. Distribution was tested by the Mann–Whitney U Test and the Shapiro–Wilk Test. The Kaplan–Meier survival curve was used to analyze implant survival. Descriptive analysis was performed to determine the mean and standard deviation (SD).

The approval for this study was obtained from the ethics committee of the Medical University of Vienna (approval number 2022/2018).

## 3. Results

### 3.1. Patient Demographics

A total of 64 patients were implanted with the Bb; five of these patients were implanted bilaterally (*n* = 69 Bb devices). Patient demographics including types of hearing loss are presented in Table 1. Five patients were lost to follow-up (all implanted unilaterally); therefore, only 64 implants were analyzed. The mean age of patients was 38.3 years (range 5.2–80.4 years). The mean follow-up was 27.1 months (range: 0.2 months–6.3 years). On average, implants were used for 25.9 months (range: 0.2 months–6.3 years). Fifty-seven implants (89.1%) were still in use at the end of the observation period.

### 3.2. Surgical Procedure

The data on explantations or revision surgeries are presented in Table 2. The overall complication rate was 9.4% (6/64). Ten out of 64 implants (15.6%) were positioned in a radical cavity. Three of these patients (33%) experienced a postoperative infection in the radical cavity which resulted in skin dehiscence. The other three explantations or revisions were due to wound dehiscence, device damage and lack of benefit.

Three cases of skin dehiscence occurred in patients where the implant was placed into the radical cavity. One patient suffered from isolated dehiscence of the wound. This patient had been initially provided with two BAHAs and two ear epitheses at the age of four which had caused chronic skin irritation. One BAHA was then replaced with a Bb after 15 years of use. After several years, the Bb also had to be removed, as the titanium containment had damaged the previously thinned skin area that had been required for the BAHA implantation.

There was only one device failure in a patient who was a youth league professional soccer player and suffered multiple head traumas which probably damaged the device. This patient was reimplanted with another Bb device, with subsequently good auditory results. 

**Figure 1:**
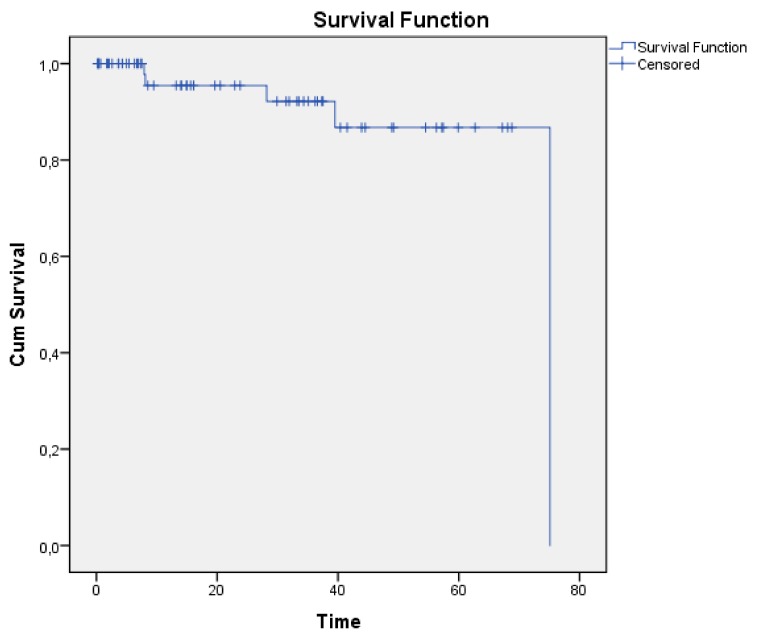
Kaplan–Meier implant survival graph for medical complications. Time; time in months.

The rate of implant loss for technical defects due to external damage was 1 per 72 follow-up years. We calculated this by adding together the total duration of follow-up time, with non-usage not considered as implant loss. Figure 1 shows the implant survival rates for medical complications.

### 3.3. Audiological Assessment

**Figure 2:**
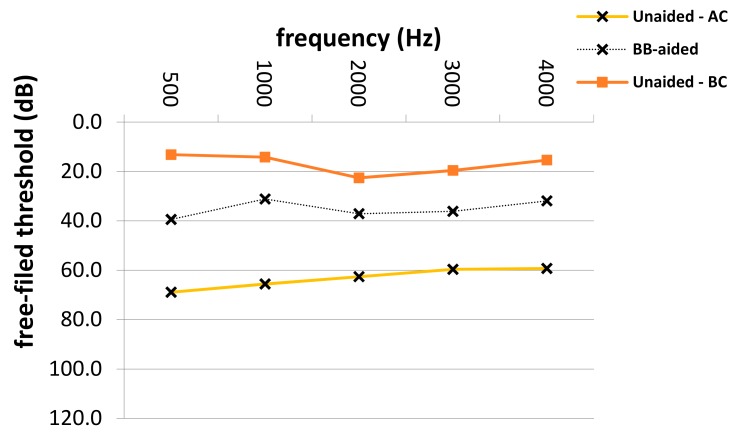
Aided and unaided free-field thresholds in patients with atresia. Hz: Hertz; dB: Decibel; Bb-aided; Bonebridge aided free-field audiometry thresholds, Unaided–AC; unaided air conduction audiometry thresholds, Unaided–BC; unaided bone-conduction audiometry thresholds.

**Figure 3:**
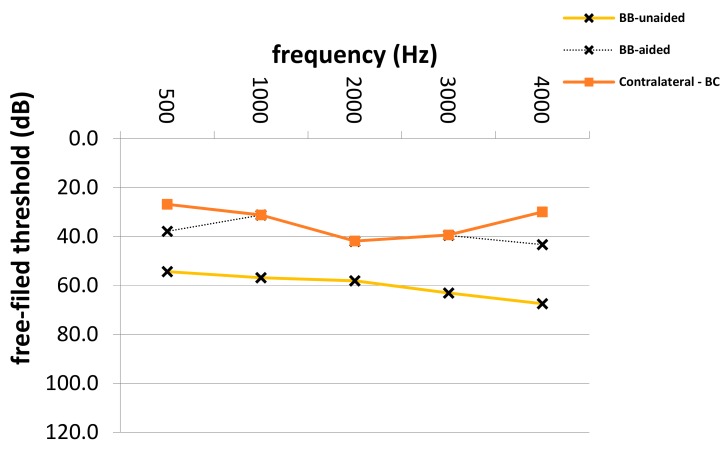
Aided and unaided free-field thresholds in patients with SSD. SSD; single-side deafness, Hz; Hertz, dB; Decibel, Bb-aided; Bonebridge aided free-field audiometry thresholds, Bb-unaided; Bonebridge unaided free-field audiometry thresholds, Contralateral–BC; unaided bone-conduction audiometry thresholds. In spite of the fact that an ear muff was used, these thresholds represent the bone conduction of the contralateral ear due to hearing loss etiology in these patients (SSD).

**Figure 4:**
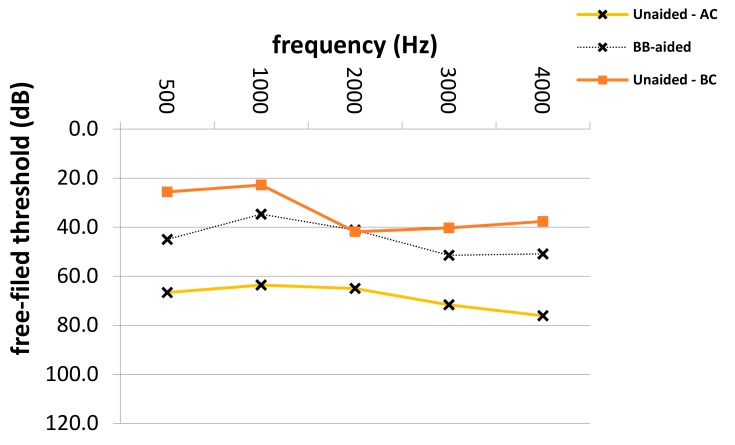
Aided and unaided free-field thresholds in patients with combined hearing loss. Hz; Hertz, dB; Decibel, Bb-aided; Bonebridge-aided free-field audiometry thresholds, Unaided–AC; unaided air conduction audiometry thresholds, Unaided–BC; unaided bone-conduction audiometry thresholds.

The average SF PTA4 threshold improved significantly from 65.4 to 38.9 dB (*p* = 0.0001), with an FG of 26.5 dB SPL (± 3.1 dB SD) in patients with atresia and combined hearing loss (see Table 3). Frequency-specific SF thresholds in patients with different types of hearing loss are depicted in Figure 2, Figure 3 and Figure 4. The highest mean FG was observed at 1 kHz (32.2 dB), followed by hearing gains at 4 and 0.5 kHz (26.6 dB and 26.4 dB, respectively). The lowest hearing gains were at 2 and 3 kHz (24.8 and 22.3 dB, respectively). Improvement in SF thresholds was statistically significant at each frequency (*p* = 0.0001 each). The effective gain in patients with combined hearing loss was −11.0 dB.

Mean speech intelligibility, tested with the WRS, improved significantly (*p* = 0.0001), from 13.3 % to 65.4 % (an improvement of 52.1 %). This rate of improvement was significant (*p* = 0.0001) for all three types of hearing loss (see Table 3).

## 4. Discussion

To the best of our knowledge, this is the largest study that systematically evaluates audiological, as well as technical and medical outcomes including the mean length of implant usage, medical and technical complications, revisions, reimplantations, explantations, implant survival and implant loss for added up follow-up years of the Bb in a single tertiary referral center. We provide evaluation of all 64 implanted patients. To date, there have been no reports on Bb user-rates in long-term follow-up studies. Our data showed that 89.1% of the implants were still in use at the end of the observation period, with a mean follow-up of 2.3 years. 

As shown in Table 2, adverse medical or technical complications occurred in 6 out of 64 patients implanted with Bb devices (9.4%). Five devices (7.8%) were explanted without reimplantation with another hearing implant. Device failure occurred in only one case (1.6%), which was probably caused by external damage. Two unilaterally-implanted patients (3.1%) were explanted and provided with a cochlear implant due to progressive hearing loss. These two cases were not included in the complication rates. 

Complications occurred statistically significantly more often in patients where the Bb was placed in a radical cavity (*p* = 0.015). In all of these cases, an infection of the radical cavity resulted in skin dehiscence over the implant. Therefore, the implants had to be removed in all three patients. One patient suffered from comorbidities such as diabetes and obesity. Another patient who had a history of five recurrent cholesteatomas initially had a wound dehiscence and then complained about persisting pain over the implant which was caused by a radical cavity infection, resulting in explantation. Wound infection and chronic inflammation of the subcutis led to explantation in one patient who also had a past medical history of a modified radical cavity. 

The fact that all of these patients had histories of radical cavities following multiple cholesteatomas underlines the importance of avoiding placement of the Bb in radical cavities, with the goal of circumventing complications and revisions. In these types of patients, the BC-FMT should not be anchored as usual in the area of the mastoid, as the contact to the external auditory canal will cause infection and inflammation. Instead, a retrosigmoid or middle fossa approach could be used, although this might not be possible in some cases. In our patients, the surgeon chose a mastoid placement due to the delicate anatomy of the thin bone. None of these patients were straightforward cases, with multiple prior surgeries and complex anatomies complicating their implantation process. They received their implant as a rescue approach to restore hearing. Today, a new version of the Bb is available where the depth measured from the skull surface is reduced to 8mm. With this implant, a retrosigmoid placement should be possible even in difficult cases.

One other patient had been part of the first clinical trial to evaluate the indication criteria for the Bb and was subsequently found to be outside of the indication criteria (45 dB maximum SPL BC threshold from 0.5 to 3 kHz) at 1 kHz, with a threshold of 50 dB SPL BC. This patient had to be explanted due to a complete lack of benefit. So far, only a few studies with a relatively small number of patients have assessed the surgical and technical outcomes with the Bb. One short-term analysis of six patients during a 6-month observation period reported no major complications [12]. Baumgartner et al. [13] published a 3-month follow-up study on 12 Bb-implanted patients, with an overall device-related complication rate of 8.3%. No device failure was reported. Two non-device-related complications (16.6%) were noted. One patient had a herpangina infection, which was satisfactorily treated. Another case involved an earlobe ischemia in a patient undergoing an outer ear reconstruction parallel to the Bb implantation. Tang et al. [10] assessed surgical outcomes in 20 patients implanted with a Bb. Only two cases (10%) of postoperative skin infection occurred, with both recovering within a week after local and oral antibiotic therapy. No other complications were reported. The complication rate in our study is slightly higher than those noted in the above-mentioned reports. However, these other studies reported on much smaller sample sizes than our study (59 vs. 6, 12 and 20 patients, respectively).

Typically, percutaneous bone-anchored hearing aids have shown high rates of postoperative complications. One study on 41 BAHA (percutaneous bone-conduction implant) patients reported a skin complication rate of 29% [14]. Another author noted a BAHA technical device failure rate of 3.2% [15], which is higher than the rate in our cohort. A slightly higher implant loss of 3.8% was noted by another author [16]. Ricci et al. [17] analyzed 49 BAHA devices that resulted in a 6.1% complication rate. Fussey et al. [18] noted that 35% of 52 patients implanted with BIA300 required revision surgery. 

The implant loss for added up follow-up years is an outcome parameter which indicates the number of devices lost due to technical complications [19,20]. Given the fact that the follow-up period of patients implanted with the relatively new Bb in our study is fairly short, the result of one implant loss for 72 follow-up years should be quite satisfying (mean follow-up: 2.3 years).

### Audiological Evaluation

The mean overall FG in patients with atresia and combined hearing loss was 26.5 dB, which is slightly lower than results found in the literature (26.9 dB [6] and 29 dB [7] to 30 dB [10]).

Compared to other studies, our data revealed a slightly favorable mean FG (28 dB vs. 19.2 [21], 25.2 dB [22]) in patients with atresia. The mixed-hearing loss group in our study had a mean FG of 24.1 dB, compared to two authors reporting on slightly higher FG values of 28 dB [23] and 33.5 dB [9]. The hearing gain that needs to be provided by the device in patients with SSD depends only on the size of the head. The effect of the head shadow is most dominant in high frequencies (>4 kHz). Unfortunately, these were not routinely measured, and were therefore not addressed in this manuscript.

The mean overall preoperative and postoperative word recognition score on the Freiburger monosyllabic test for all patients at 65 dB SPL was 13.3% versus 65.4%, with a statistically significant mean improvement of 52.1% (*p* = 0.0001). Our results correspond to those reported by other authors [9,22,23].

## 5. Conclusions

The present data confirm that the Bonebridge provides safe and effective long-term hearing rehabilitation. In conclusion, the Bb has proven to be a safe system as shown by very moderate complication and revision rates. The device failure rate was very low and caused by external trauma. The efficacy of the implant was confirmed by the audiological evaluation. Meticulous patient selection, mainly with regard to co-morbidities and proper surgical technique, seems to be a crucial factor in reducing complications. Furthermore, individual anatomy, especially in children, and a history of chronic otologic disease should be taken into account when planning a Bb implantation for hearing rehabilitation.

## Figures and Tables

**Table 1:** Patient demographics.

Indication	Mean Age, Years	Age range, Years	Male/Female, n	Male/Female, %	n/%
Atresia	25.1	5.2–56.9	19/11	63.3/36.7	30/43.5
SSD	45.6	7.0–72.9	8/12	40.0/60.0	20/29.0
Comb HL	51.4	23.3–80.4	10/9	52.6/47.4	19/27.5
Total	38.3	5.2–80.4	37/32	53.6/46.4	69/100

**Table 2:** Revisions and explantations.

Complication	Etiology	Therapy	*n*/%	*n*/%
Skin dehiscence	Implanted out of anatomical indication criteria (Radical cavity)	Explantation	3/4.7	5/7.8
Wound dehiscence	Implanted out of anatomical indication criteria (previous BAHA)	Explantation	1/1.6	
Lack of benefit	Implanted outside of indication criteria	Explantation	1/1.6	
Device damage	Recurrent head trauma	Reimplantation	1/1.6	1/1.6
No complications			58/90.6	58/90.6
				64/100

**Table 3:** Functional gain (FG) and mean WRS improvement.

Indication	Mean FHG (dB)	*p* Value	Mean Hearing Gain (%)	*p* Value
Atresia	28 ± 3.8	<0001	57.5 ± 7.8	<0001
SSD	/	/	49.0 ± 6.6	<0001
Comb HL	24 ± 3	<0001	44.4 ± 11.1	<0001
